# The Effect of the Gaseous Environment on the Electrical Conductivity of Multi-Walled Carbon Nanotube Films over a Wide Temperature Range

**DOI:** 10.3390/ma13030510

**Published:** 2020-01-21

**Authors:** Dawid Janas, Krzysztof K. Koziol

**Affiliations:** 1 Department of Chemistry, Silesian University of Technology, B. Krzywoustego 4, 44-100 Gliwice, Poland; 2 Department of Materials Science and Metallurgy, University of Cambridge, 27 Charles Babbage Rd, Cambridge CB3 0FS, UK; kk292@cam.ac.uk; 3 Department of Transport and Manufacturing, Cranfield University, College Road, Cranfield MK43 0AL, UK

**Keywords:** carbon nanotubes, thin films, electrical conductivity

## Abstract

The surrounding gas atmosphere can have a significant influence on the electrical properties of multi-walled carbon nanotube (CNT) ensembles. In this study, we subjected CNT films to various gaseous environments or vacuum to observe how such factors alter the electrical resistance of networks at high temperatures. We showed that the removal of adsorbed water and other contaminants from the surface under reduced pressure significantly affects the electrical conductivity of the material. We also demonstrated that exposing the CNT films to the hydrogen atmosphere (as compared to a selection of gases of inert and oxidizing character) at elevated temperatures results in a notable reduction of electrical resistance. We believe that the observed sensitivity of the electrical properties of the CNT films to hydrogen or vacuum at elevated temperatures could be of practical importance.

## 1. Introduction

The discovery of carbon nanotubes (CNTs) in 1991 [1] brought a new player into the field of engineering able to surpass many technological limitations of classical materials. Due to their unique nanostructure, which translates into impressive electronic [2,3], thermal [4], and mechanical [5] properties, an unprecedented level of performance has been observed for individual CNTs. As a consequence, they have become one of the most promising candidates for many applications ranging from nanoactuators [6], nanobatteries [7], nanosensors [8] to other forms of nanodevices [9,10,11]. Breakthrough in the production of CNT ensembles beyond the laboratory scale from the liquid [12] or solid medium [13,14] and further research advances have eventually led to the creation of real-life scale CNT applications such as heaters [15], cloaking systems [16] or hologram emitters [17].

Interestingly, these 1D conductors have revealed that their electronic character is quite sensitive. The exposure of CNT ensembles to a number of chemical species has been found to have a strong influence on their electrical properties [18]. Because of this, and the fact that their surface area reaches up to 1000 m^2^·g^−1^ [19], there is a particular interest in the application of CNTs in the field of gas sensing. There have been numerous reports on using CNTs as sensors of hydrogen [20], oxygen [21], methane [22], and many more [23,24,25,26], a prevailing number of which, however, operate at room temperature. Since a wide spectrum of processes in the industry actually takes place at elevated temperatures, it is of high importance to develop sensors able to work under non-standard conditions.

In this work, we used the direct-spinning method to produce multi-walled CNT films in a single step [13], which were then engaged as resistive heating elements as previously reported [15]. This time, however, they were exposed to various gaseous environments while kept at high temperatures. The motivation was to probe what is the influence of particular gaseous species on the electrical properties of CNT films under non-ambient conditions. For this purpose, we chose high-vacuum, as well as a selection of inert, oxidizing, and reducing environments. The results showed that interaction with the surrounding gas makes an impact on the mobility of charge carriers within the explored temperature range. Firstly, we quantified the influence of the vacuum level on the electrical resistance of CNT ensembles at 100 °C. Then, we gauged the change in electrical resistance when the CNT films were exposed to different gaseous environments within room temperature at a −300 °C temperature window. Finally, we observed how the elimination of oxidizing species allows for strong and stable incandescence from these resistively heated CNT films.

## 2. Materials and Methods

### 2.1. Synthesis of CNT Films

**Figure 1:**
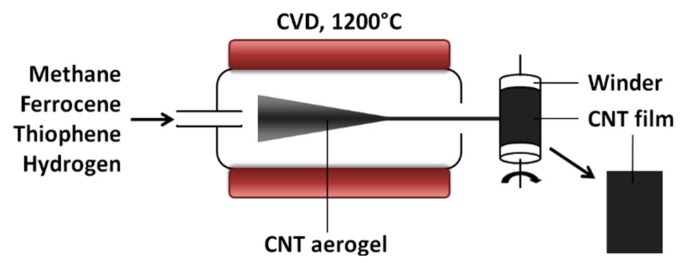
Experimental setup for the synthesis of carbon nanotube (CNT) films by the direct-spinning method.

Methane was subjected to chemical vapor deposition (CVD) catalyzed by ferrocene (p.a.; Sigma-Aldrich, Gillingham, UK) and promoted by thiophene (p.a.; Sigma-Aldrich, Gillingham, UK) inside of a vertical reactor kept at 1200 °C under hydrogen. Continuously produced aerogel that formed inside was drawn directly out from the reactor and transferred onto a fast-spinning winder to yield 10 μm thick CNT films made up of multi-walled CNTs as previously described (Figure 1) [13,27].

**Figure 2:**
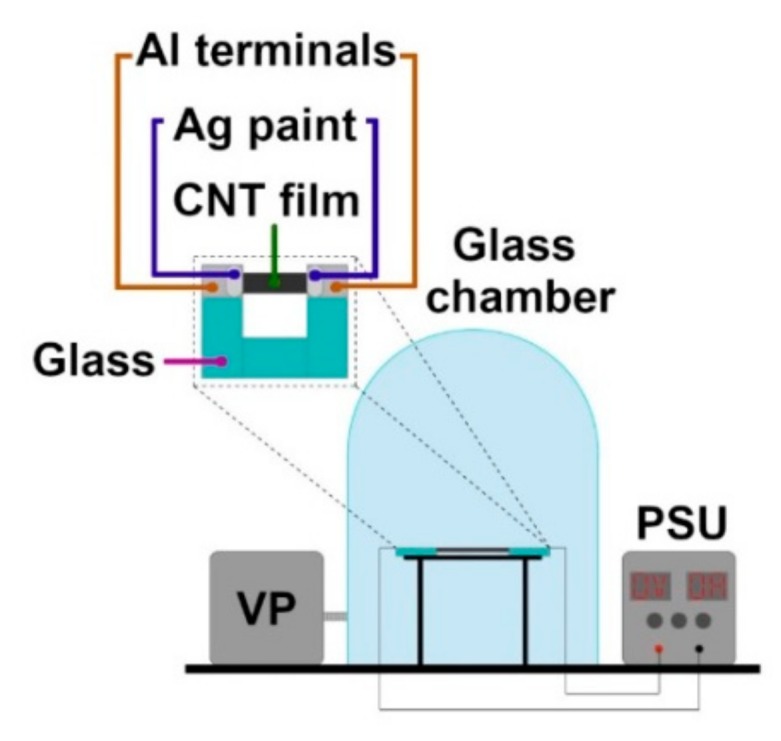
Experimental setup for testing of CNT films (sample holder magnified). VP—vacuum pump; PSU— power supply unit.

The material was then cut with a razor blade into 10 mm × 40 mm specimens, peeled off the substrate, and transferred onto custom-designed sample holders (Figure 2). The U-shaped holders were made of glass and equipped with Al tape electrical terminals between which the CNT films were placed. Ag conductive paint was used to minimize the possible effect of contact resistance between nanocarbon and the Al terminals through which current was delivered.

### 2.2. Assessing the Electrothermal Properties

Previously reported methodology [15] was adopted to find the dependence between employed electric power and temperature. In short, the CNT films were biased in the air with DC (TTi 120 H power supply, TTi, Huntingdon, UK) and the temperature of the ensemble was recorded with a pyrometer (Impac IPE 140, Advanced Energy Industries, CO, USA). At the beginning of the study, the accuracy of the temperature measurements acquired in a non-contact mode was cross-checked with a thermocouple and the outcomes were in accordance with each other. Stepwise change of bias voltage while measuring the temperature was conducted in the course of two consecutive runs from room temperature up to 300 °C. The first run was regarded as a pretreatment step, during which heat-assisted evaporation of absorbed species takes place and causes a quasi-permanent change in resistance [28]. The values of electric power and the corresponding temperatures obtained from the second run in such a way enabled us to predict the temperature of the CNT films inside the glass chamber. An online temperature measurement was not possible inside of the enclosure wherein they were subsequently examined under various gaseous atmospheres.

### 2.3. Electrical Measurements under Non-Ambient Conditions

All samples evaluated in this work were prepared as specified before. They had an area of 10 mm × 40 mm and were pretreated as described above to remove as much adventitious contamination as possible coming from the synthesis stage and storage in the ambient. For these investigations, sample holders with CNT films were placed on the stage, crocodile clips leading to the power supply unit were connected to the Al tape electrical terminals, and then the glass chamber was properly sealed (Figure 2).

#### 2.3.1. Vacuum Experiments

In the first experiment, a turbo vacuum pump was turned on while the CNT film was biased at 9 V, which corresponded to 100 °C. We monitored the effect of pressure on the value of the current by bringing the vacuum to a certain level and maintaining it at this point until the electrical read-outs stabilized. We decreased the pressure in steps eventually reaching 5 × 10^−6^ bar.

**Figure 3:**
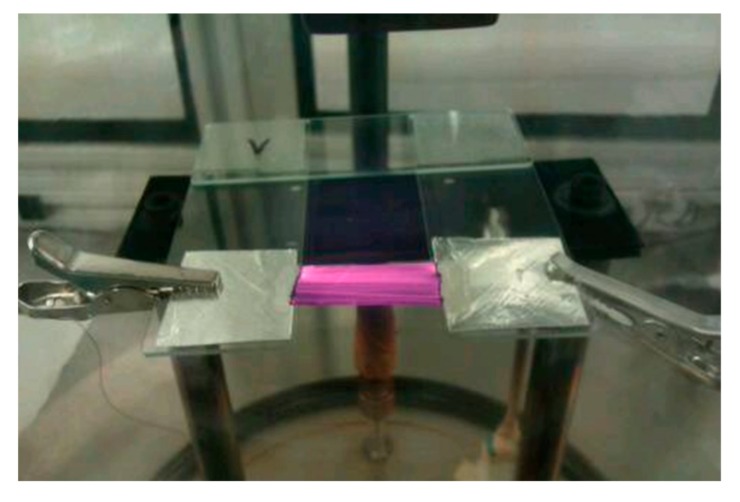
Incandescence from the CNT film at 67.5 W of delivered electric power.

In the second experiment, we probed the influence of vacuum on current–voltage characteristics of the CNT films in the extended temperature range. In the beginning, the electrical behavior was tested in the air up 300 °C, so as to prevent oxidation. Then, we pumped the system down to 2 × 10^−6^ bar and repeated the I–V measurements in the oxygen-deprived environment on the same CNT film. This time, however, we ramped up the bias voltage to the limits of the DC power supply. Notable incandescence from the sample was observed during this time (Figure 3). Lastly, we switched off the power supply unit and filled the chamber with air again. Then, we increased the bias voltage stepwise up to the failure point in air, which occurred at 461 °C.

#### 2.3.2. Artificial Atmosphere Experiments

Once we evacuated the chamber and reached 2 × 10^−6^ bar, we bled methane, acetylene, oxygen, ammonia, nitrogen, argon, or hydrogen into the system up to the point when the pressure gauge indicated 2 × 10^−2^ bar. I–V data were collected on the samples again from room temperature up to 300 °C and compared with the behavior of the samples examined in air. We used the relation of employed electric power to the temperature of the material (described in Section 2.2) to determine the temperature of the CNT films inside the chamber.

### 2.4. Characterization

BET adsorption/desorption isotherms (Tristar 3000, Micromeritics Instruments Corporation, GA, USA) were recorded using N_2_, wherein P is the actual pressure and P_0_ is the saturation pressure of N_2_ at 77 K. Prior to the measurement, the sample was outgassed at 140 °C overnight. Ten milligrams of the material were used for the measurement.

Scanning electron microscopy (SEM; JEOL6340 FEG SEM; Tokyo, Japan) was employed to observe the material microstructure and probe for the presence of carbonaceous adulterants before and after the electrothermal treatments.

Raman spectroscopy (Renishaw RM2000, λ = 633 nm, HeNe 2 mW; Wotton-under-Edge, UK) was used to acquire the intensity of the defect-induced band (D) as well as that of the band of vibrations of graphitic structures (G). I_D_/I_G_ ratio was employed for analysis, which is a common way to gauge the level of structural perfection of C-sp^2^ lattice in nanocarbon materials.

## 3. Results

### 3.1. Experiments in Vacuum

**Figure 4:**
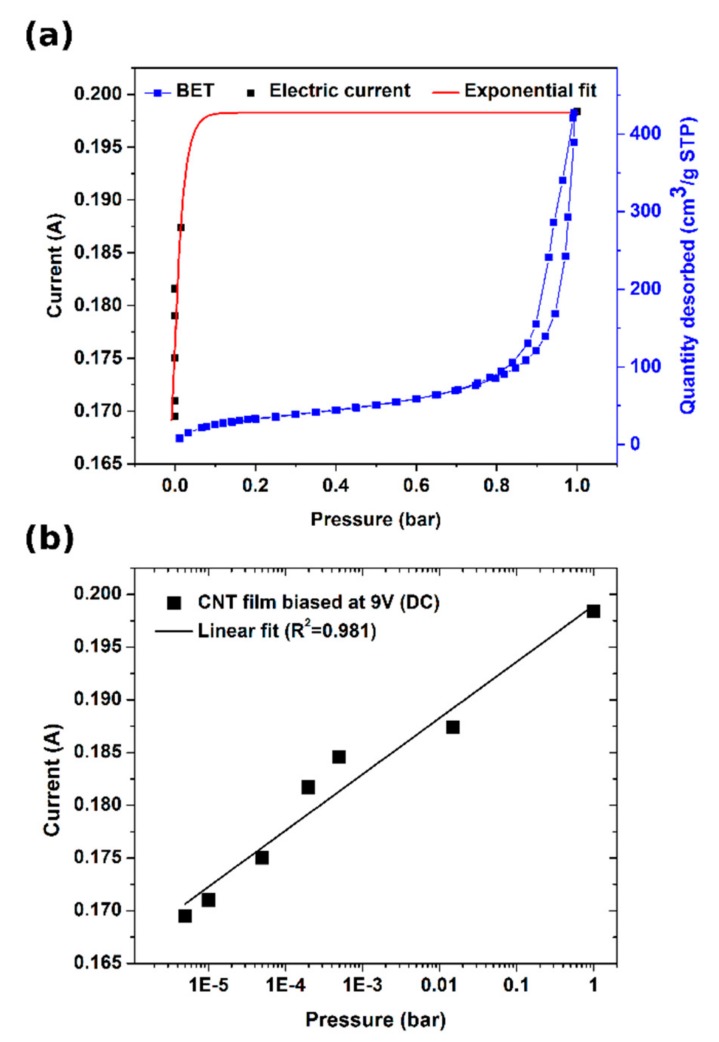
(**a**) Effect of pressure on the electrical conductivity of CNT films correlated with N_2_ desorption isotherm. (**b**) Electrical conductivity of CNT films as a function of pressure.

First, we probed the porous nature of our CNT films by using a nitrogen adsorption–desorption method at 77 K (Figure 4a). The material proved to have mostly meso- and macropores as expected from a CNT aerogel [15,29]. Its isotherm can be classified between II and IV types [30,31].

In the enclosed plot, one can note that a monolayer of N_2_ is reached rapidly, then there is a plateau, at which multi-layer formation starts and, finally, hysteresis appears, which is indicative of capillary condensation. To get to know how various regimes of vacuum affect the conductivity of the CNT films, we measured their electrical properties at diminished pressure. Figure 4b shows that the current is in logarithmic relation with pressure and decreases with improving the quality of the vacuum. It can be explained by the desorption of dopants present on the surface, which in the ambient conditions enhance the conductivity of CNT networks. The doping species that could come off the CNT films readily at such a low pressure are water [32,33] and oxygen [34] molecules. Their removal is particularly difficult as they are confined within the framework and structure of the CNT films and require a significant pressure difference to desorb them fully. A study by Chaban et al. [35] shows that water bound to the CNT network can, in fact, have elevated boiling point, so it is very difficult to fully get rid of it. We measured an 18% increase in resistance as the chamber with a CNT film was evacuated from ambient pressure down to 5 × 10^−6^ bar at 100 °C. Since the increase in resistance with the level of vacuum did not seem to decelerate under the evaluated conditions, we may suspect that the CNT films are still doped to some extent. Finally, we brought the pressure down to 2 × 10^−6^ bar and, once the electrical properties stabilized, we started increasing the bias voltage up to the limit of the DC power supply eventually reaching 67.5 W (77 V × 0.877 A). In these settings, the CNT film was glowing substantially due to the incandescence from the material [36] (Figure 3).

**Figure 5:**
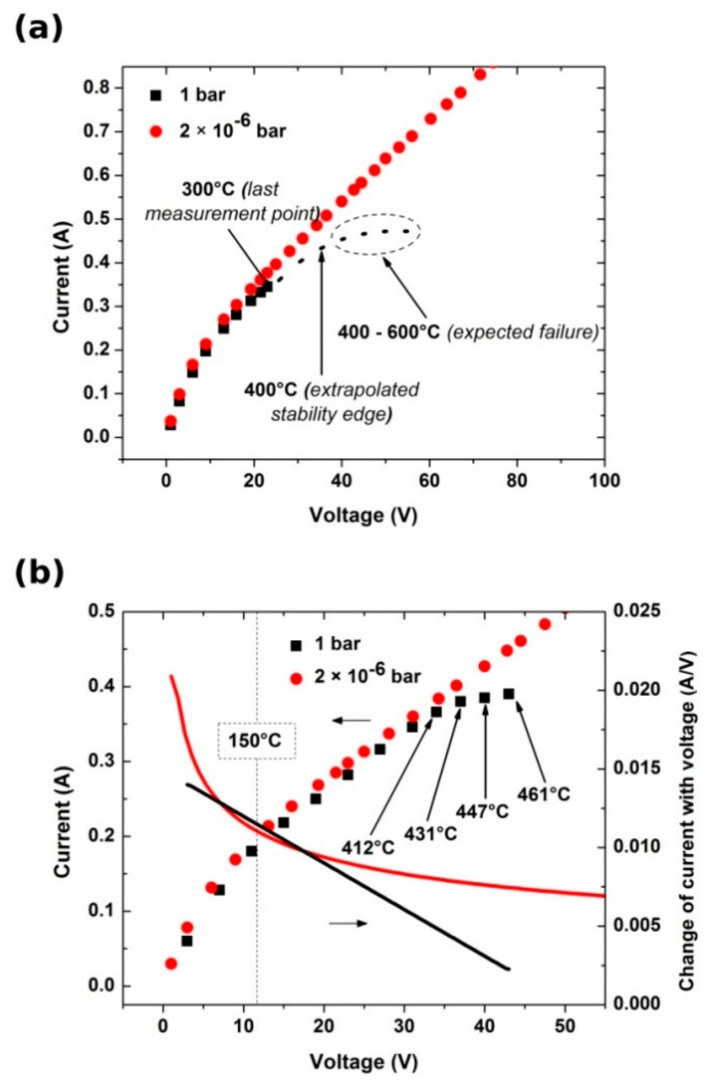
Effect of oxygen elimination on the electrical conductivity and thermal stability of CNT films (**a**) up to 300 °C and (**b**) within the extended temperature range.

As shown in Figure 5a, in the beginning, CNT films exhibit non-Ohmic behavior up to 150 °C. In this regime, heat-assisted removal of residual water as well as aliphatic and aromatic contaminants [37,38] from the synthesis stage takes place. These species readily deposit onto the nanocarbon due to relatively high porosity of the material and interfere with the observed electrical and/or surface properties [38]. 

As reported before, the evaporation of these species can give CNT films with Ohmic behavior in consecutive runs [15].

Furthermore, in the case of CNT films heated in air, we entered the edge of the thermal stability region beyond 300 °C, which extends up to about 400 °C [39]. At this point, we can expect the electrical breakdown of the most conductive metallic CNTs because of high current densities [40,41]. Further increase in delivered electric power and hence the rise in temperature of the CNT films usually results in rapid oxidation at the temperatures between 400 °C and 600 °C, which breaks the electric circuit. The actual failure temperature is dependent upon the composition of the CNT films. The number of constituting walls and the degree of structural perfection both have a strong impact on the electrothermal performance. It is commonly known that the least thermally stable CNT ensembles are composed of defected single-walled CNTs. In our case, the use of multi-walled CNTs and evacuation of the chamber suppressed this issue. Because of the lack of contact of the CNT films with air, they continued to exhibit linear I–V characteristics in the high-temperature regime. Closer investigation revealed that the increase of current with bias voltage under vacuum approaches a steady pace—the first derivative of the I–V curve shown in Figure 5b tends to a constant (in contrast to that of the sample heated in air). 

**Figure 6:**
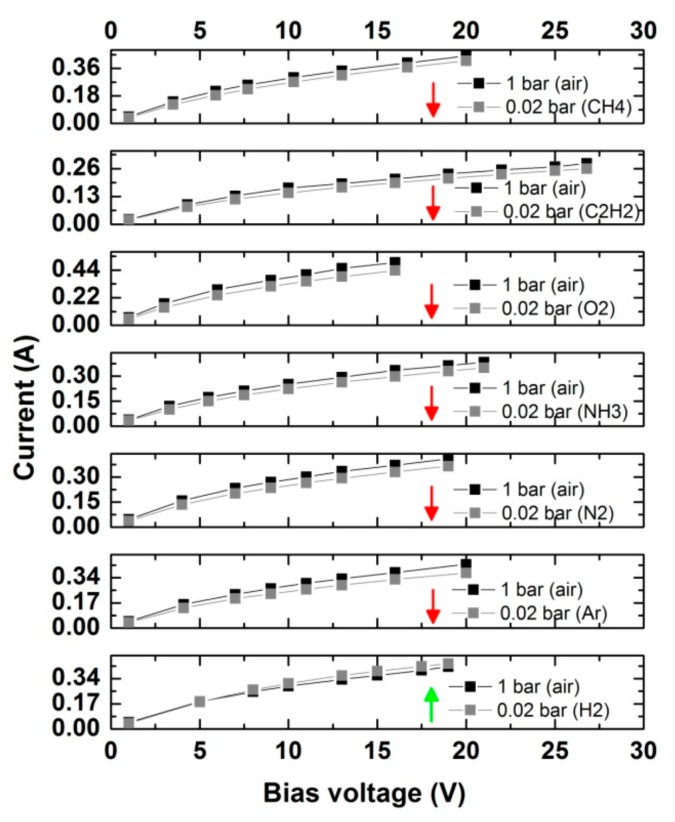
Gas effect on CNT films and their I–V characteristics (red arrow and green arrows indicate a decrease and increase in conductivity, respectively).

Our next aim was to observe how different gaseous atmospheres affect the electrical properties of CNT films. The chamber was evacuated down to 2 × 10^−6^ bar and then we bled selected gases up until the pressure of the artificial atmosphere had reached 2 × 10^−2^ bar. We monitored the I–V characteristics of the CNT films between room temperature and 300 °C in these conditions (Figure 6). An increase in resistance was observed in all of the cases except when the CNT film was exposed to hydrogen. Absorption of these species on the surface improved the mobility of the charge carriers. 

**Figure 7:**
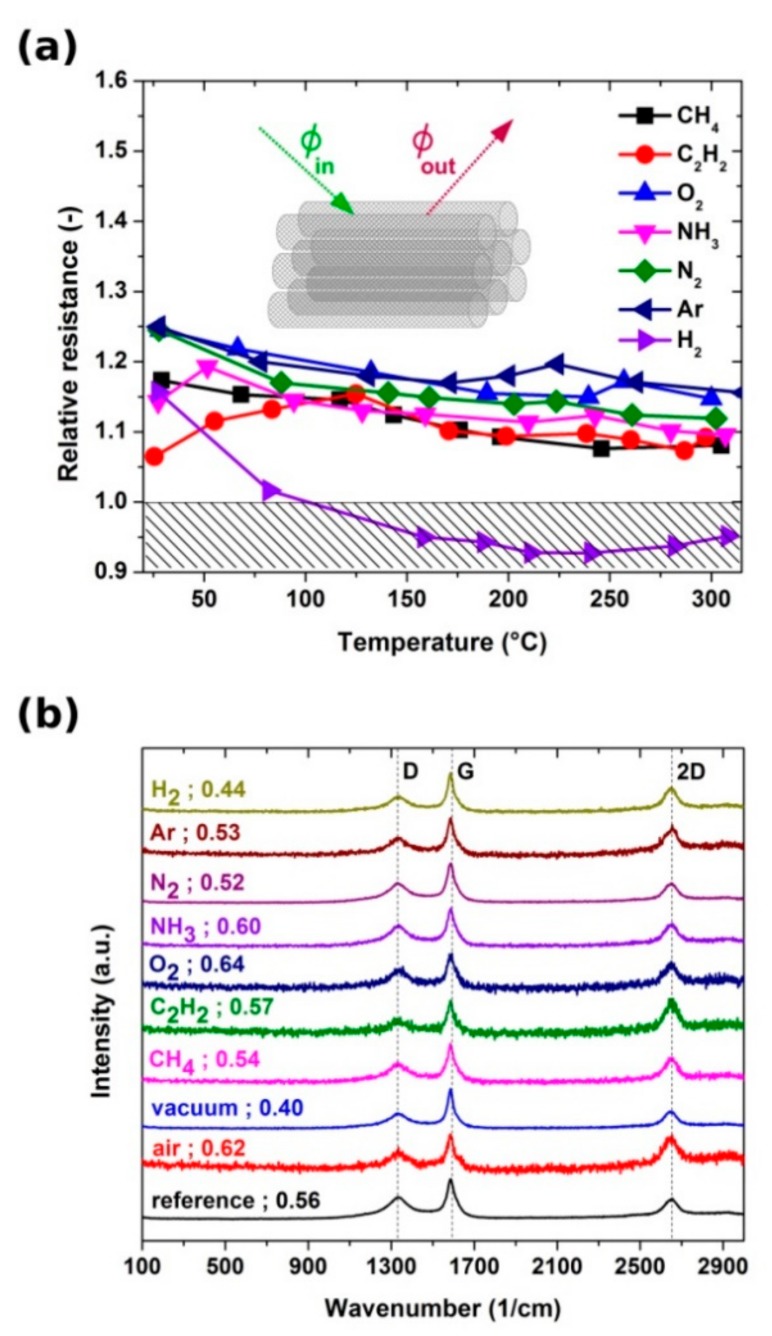
(**a**) Surrounding gas effect on the electrical conductivity of CNT films up to 300 °C. (**b**) Raman spectra of CNT films: as-made and after the electrothermal treatments in various environments.

To analyze this effect in more detail, we normalized the resistance of the samples in various environments (by taking the I–V characteristics acquired in air as a reference) and compared the results (Figure 7a). We can see two types of behavior, i.e., a population of samples with increased resistance (exposed to methane, ethylene, oxygen, ammonia, nitrogen, and argon) and the sample for which the resistance decreased (exposed to hydrogen). The behavior of the former group of samples can be explained by the evaporation of water during the evacuation of the chamber. As it desorbs from the CNT films, its doping action fades, and thus we observe an upshift in resistance by about 15% over the whole temperature range (close to 18% observed in Section 3.1 for results obtained in vacuum justified by the same effect). It appears that, under such conditions, these gases do not affect the electrical properties of the material.

Hydrogen presence, on the other hand, overcame this phenomenon and gave a decrease in resistance. As depicted in the inset, there is always a net flux of gas, according to Fick’s first law. Hydrogen diffuses into CNT networks very well [42,43] (diffusion coefficient is much higher than that of other employed gases), so we can expect that it will readily penetrate the structure of our CNT film, which is of the aerogel nature. It also has to be taken into consideration that nanocarbon strongly interacts with hydrogen, and hence they have been considered for a long time as a promising hydrogen storage material [44].

The hydrogen sensing properties of CNTs have been well-explored, but in most cases while covered with Pd particles at room temperature [45,46,47,48,49]. The reason for the addition of metal nanoparticles for such purposes is that hydrogen creates a hydride layer on the surface of Pd, which spills over the CNTs [46,47]. Our neat CNT films experienced a maximum sensitivity toward hydrogen at about 225 °C, at which point the electrical resistance decreased by 7%. CNTs themselves are capable of causing hydrogen dissociative adsorption and the process can be auto-accelerating [50]. There is an interplay between the role of temperature on net diffusion flux of gas as well as on the ability of hydrogen molecules to dissociate. The combined effect herein is a favorable decrease in resistance. One also has to keep in mind that, in fact, hydrogen affects the resistance to a larger degree because it counteracts the increase of resistance caused by the desorption of water from the CNT films under vacuum (the samples were first exposed to vacuum and then put in contact with hydrogen inside the glass chamber).

Since CNT films kept at elevated temperatures under various gaseous environments can experience a change in chemical composition or microstructure [51], we characterized the samples before and after the treatment. Examination by Raman spectroscopy probed for the first type of modifications (Figure 7b). Exposure of the CNT films to inert gases (N_2_, Ar) or hydrocarbons (acetylene, methane) did not cause appreciable alteration to the I_D_/I_G_ ratio and small differences may be accounted for by slight inhomogeneity of the material. That was expected from the chemistry of the material as neither CNTs nor present adulterants should react with them under these conditions. Moreover, the CNT films exposed to air or oxygen experienced an increase in the degree of disorder from 0.56 to 0.62 and 0.64, respectively. The effect can be justified by partial oxidation of non-volatile and defective carbonaceous deposits present on the surface of the material as reported previously [37]. The only other gas that caused a noticeable increase in I_D_/I_G_ ratio up to 0.60 was ammonia, which can readily react under these conditions with amorphous carbon or these contamination species present in our material [52]. An opposite effect was observed when the hydrogen atmosphere was employed. We noted a decrease in I_D_/I_G_ ratio from 0.56 to 0.44 explained by the hydrogen reduction of certain defects on the surface. Finally, the most significant improvement was noted due to the action of vacuum when the CNT film was brought up to the point of incandescence. High-temperature annealing under inert environments has a favorable influence on the degree of graphitization of the CNT assemblies [53,54], so we can expect a similar effect when resistive heating is employed for this purpose herein. Lastly, the analysis of the microstructure by electron microscopy did not visualize detectable changes done by the electrothermal treatment under various conditions (Figures S1 and S2). It can be concluded that any changes to the material detected by Raman spectroscopy take place at the level of individual CNTs while the ensemble, in general, remains intact. Deposits of amorphous carbon introduced during the synthesis persist in the CNT film despite the high-temperature treatment. This is to be expected because usually healing of CNT defects and removal of carbonaceous species is conducted at more than 2000 °C. At this point, it is also important to stress that control experiments conducted by us on annealed multi-walled CNT films revealed that the presence of residual catalysts had a negligible effect on the way the electrical conductivity of the films responded to various gaseous atmospheres. The differences between treated and untreated materials were within statistical error. Therefore, the observed sensorial action comes solely from the nanocarbon.

## 4. Conclusions

The surrounding gas atmosphere can make a significant impact on the electrical conductivity of multi-walled CNT ensembles. As we subjected CNT films to various grades of vacuum, we observed that diminished pressure results in desorption of water (and other contaminants), which is correlated with an increase in their electrical resistance. This transforms the material into a conductor of an Ohmic type. What is more, the electrical properties of multi-walled CNT films are affected the most by exposing them to hydrogen, adsorption of which improves the mobility of charge carriers. We believe that the observed sensitivity of the electrical properties of the multi-walled CNT films to hydrogen or vacuum at elevated temperatures could be of practical importance. The findings can give rise to the development of a special grade of sensors for industrial applications since many processes in this field operate at elevated temperatures. Lastly, significant light emission in vacuum at high bias indicates that the CNT films can sustain notable current densities. It once again demonstrates that the elimination of the issue of contact resistance between individual CNTs in an ensemble should bring the conductivity of the network much closer to the theoretical limits obtained for individual CNTs. 

## Supplementary Materials

The following are available online at https://www.mdpi.com/1996-1944/13/3/510/s1, Figure S1: SEM micrographs CNT films as made and after the electrothermal treatment in air, vacuum, methane, and ethylene; Figure S2: SEM micrographs CNT films after the electrothermal treatment in oxygen, ammonia, nitrogen, argon, and hydrogen.
