# Supplementary material for: The Effect of the Gaseous Environment on the Electrical Conductivity of Multi-Walled Carbon Nanotube Films over a Wide Temperature Range
Source: Materials (Basel). 2020 Jan 21;13(3):510. doi: 10.3390/ma13030510 (PMC7040676; doi:10.3390/ma13030510)
Supplement: Supplementary file 1 [file materials-13-00510-s001.pdf]

Supplementary information

The effect of the gaseous environment on the electrical conductivity of multi-walled carbon nanotube films over a wide temperature range

D. Janas[1,*], K.K. Koziol[2,3]

[1] Department of Chemistry, Silesian University of Technology, B. Krzywoustego 4, 44-100 Gliwice, Poland

[2] Department of Materials Science and Metallurgy, University of Cambridge, 27 Charles Babbage Rd, CB3 0FS Cambridge, United Kingdom

[3] Department of Transport and Manufacturing, Cranfield University, College Road, MK43 0AL Cranfield, United Kingdom

[*] Corresponding author: Dawid.Janas@polsl.pl

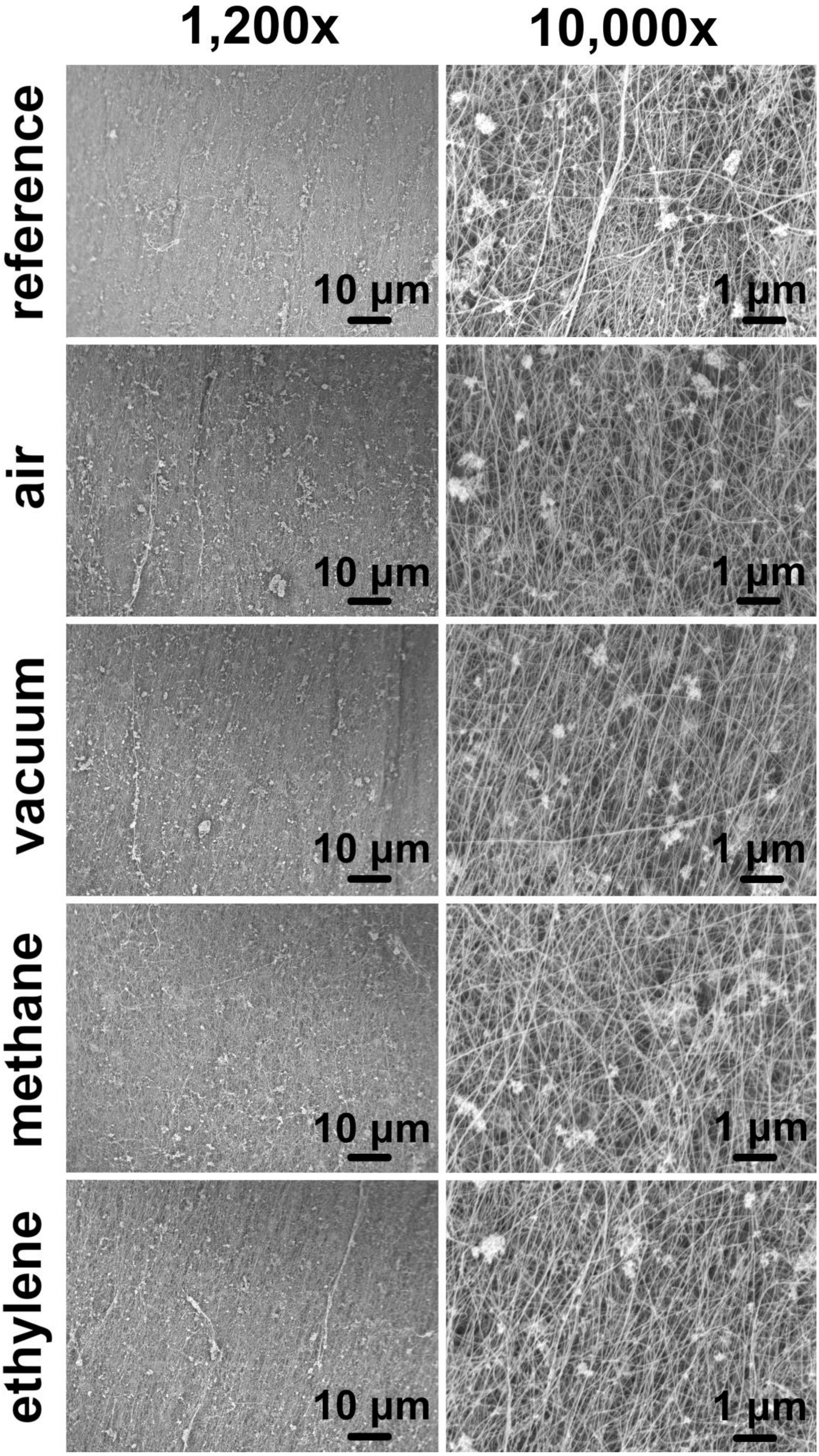

Figure S1. SEM micrographs CNT films – as made and after the electrothermal treatment in air, vacuum, methane and ethylene.

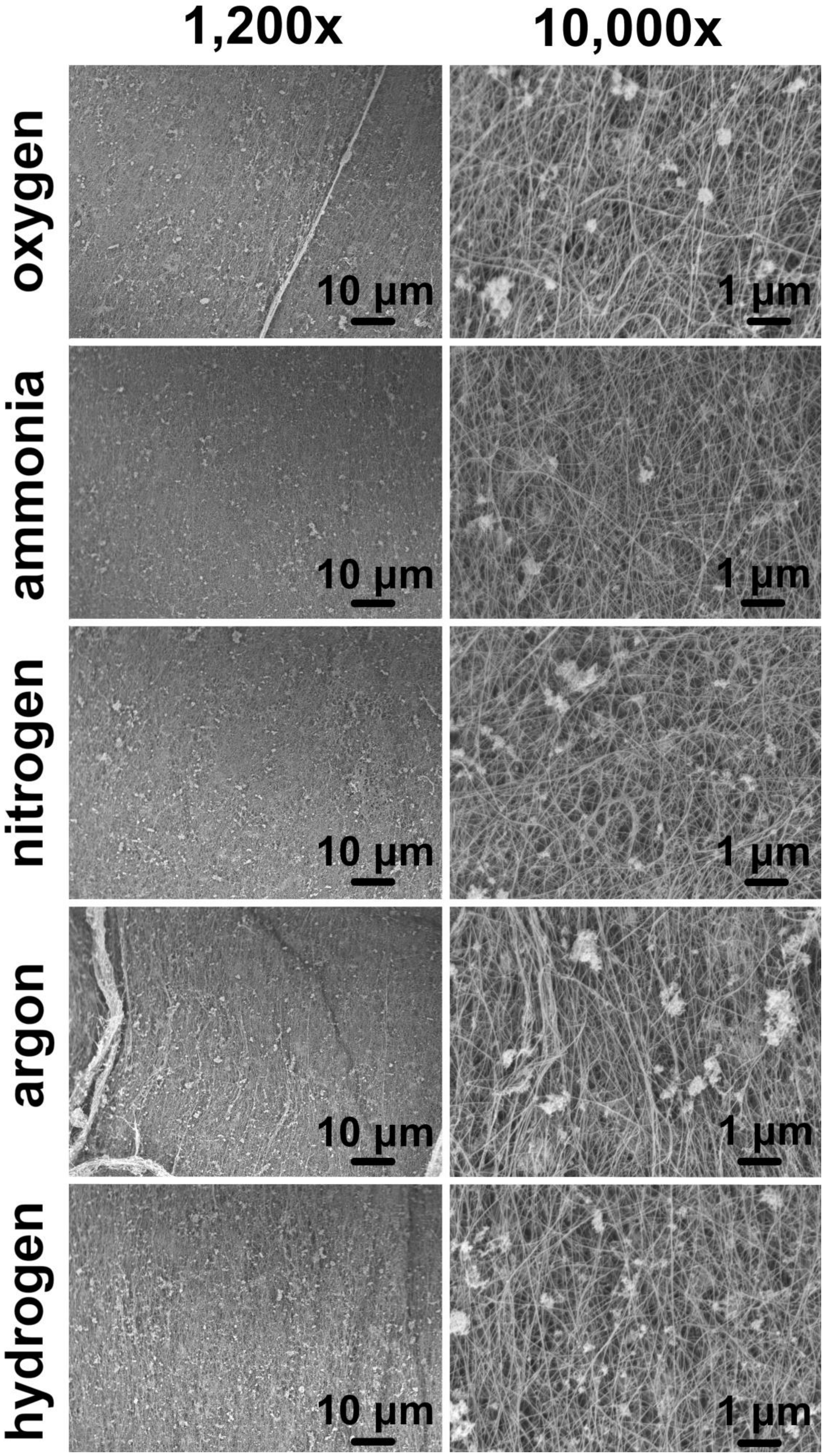

Figure S2. SEM micrographs CNT films after the electrothermal treatment in oxygen, ammonia, nitrogen, argon and hydrogen.
